# Anatomic Factors Predicting Postoperative Strabismus in Orbital Wall Fracture Repair

**DOI:** 10.1038/s41598-019-51127-7

**Published:** 2019-10-15

**Authors:** Chih-Kang Hsu, Meng-Wei Hsieh, Hsu-Chieh Chang, Ming-Cheng Tai, Ke-Hung Chien

**Affiliations:** 1 Department of Ophthalmology, Tri-Service General Hospital, National Defense Medical Center, Taipei, 114 Taiwan; 2 Department of Ophthalmology, Tri-Service General Hospital Songshan branch, Songshan, 105 Taiwan; 3 Department of Ophthalmology, Taoyuan Armed Forces General Hospital, Taoyuan, 325 Taiwan; 4 Department of Nursing, Tri-Service General Hospital, National Defense Medical Center, Taipei, 114 Taiwan; 5 Graduate Institute of Nursing, College of Nursing, Taipei Medical University, Taipei, 110 Taiwan; 6 Institute of Pharmacology, National Yang-Ming University, Taipei, 112 Taiwan

**Keywords:** Bone, Outcomes research

## Abstract

This study is aimed to determine the relationship between orbital fracture sites in each CT scan view and postoperative diplopia. Data for 141 patients of orbital wall fracture were analyzed retrospectively. One group of examiners reviewed sagittal, coronal and axial CT scans. Descriptive statistical analysis was used to assess each fracture area and its potential relationship with the occurrence of postoperative diplopia. Among the three anatomical views, sagittal sections were significantly associated with post-operative diplopia (PD) (p = 0.044). For orbital wall fractures in a single location, C1 (p = 0.015), A1 (p = 0.004) and S3 (p = 0.006) fractures were significantly related to PD. Orbital wall fractures found in more than one location resulted in a higher probability of PD in all sections:, C1 + C2 group (p = 0.010), C1 + C2 + C3 group (p = 0.005), A1 + A2 group (p = 0.034), A3 + A1 group (p = 0.005), S1 + S2 group (p < 0.001), S2 + S3 group (p = 0.006) and S1 + S2 + S3 group (p < 0.001). For combinations of two or three sections, we found that only fractures involving both coronal and sagittal sections led to a significantly increased risk of PD (p = 0.031). PD is the main posttreatment complication of orbital bone fracture reduction. In addition to the known myogenic cause (failure to relieve entrapment) of diplopia, both trauma and surgical manipulation can compromise ocular motor nerve function and possibly result in the development of neurogenic causes of diplopia. Careful assessment of patient symptoms (whether preoperative diplopia is present), and the location of orbital fractures (and the influence of related musculature, fat, and nerves) on CT scans are strongly related to surgical success.

## Introduction

Orbital bone fractures are a very common mid-facial injury, accounting for up to 40% of all trauma injuries in this region^1^. They occur due to direct blunt trauma that leads to a sudden increase in intra-orbital pressure and/or force transmission through the bony walls. These fractures may be categorized into two main groups, those that include the orbital rim and those in which only the walls of the orbit are involved. Trauma involving the orbital wall and adjoining soft tissue may cause significant functional and cosmetic complications, such as diplopia, ocular muscle entrapment, and enophthalmos, especially if diagnosis is delayed.

When orbital trauma is suspected, an immediate and appropriate diagnosis is crucial to allow early treatment. Computed tomography (CT) is the most common method used for imaging diagnosis in orbital traumatology. It provides information regarding the location and size of orbital fractures and possible associated soft tissue damage. Orbital CT also provides information regarding whether “emergency surgery” is required. The combination of both clinical investigation and imaging allows correct diagnosis and successful treatment of these fractures.

A few articles have discussed the relationship between CT imaging and preoperative and postoperative diplopia. However, no studies have analyzed the importance of each section of CT images for preoperative and postoperative diplopia. Here, we analyzed the probability of postoperative diplopia in each section of orbital wall fractures. The results of this analysis can remind physicians to pay more attention to the high-incidence area of diplopia before surgery. These areas should be carefully treated during surgery to avoid the possibility of postoperative strabismus.

## Methods

This retrospective study was conducted to collect data on consecutive strabismus after orbital wall fracture repair in the Tri-Service General Hospital from January 2007 to December 2016. The study protocol and supporting documents were reviewed and approved by the institutional review board (No: 1-104-05-124) of the Tri-Service General Hospital, Taipei, Taiwan. IRB was approved for this study to waive the informed consent for individual patient. IRB committee ensured our proposal to protect the privacy of the enrolled patients. The study followed the Good Clinical Practice guidelines of Taiwan and was conducted in accordance with the Declaration of Helsinki, 1964, and later revisions.

All of the patients who were selected to participate in the study had visited our hospital for orbital wall fracture repair at either the Department of Otorhinolaryngology, Department of Oral and Maxillofacial Surgery, Department of Plastic Surgery or Department of Ophthalmology and visited our ophthalmologic department for regular follow-up evaluations.

From chart review, patients were considered to have postoperative strabismus when they were diagnosed with any type of strabismus from at least one ophthalmic record by a strabismus specialist during postoperative follow up. If patients met the above inclusion criteria, their records were chosen for further analysis. Patients were eligible for inclusion in the study if they underwent fracture repair for one of the orbital walls and had at least one ophthalmic clinic visit and no history of other ocular disease, such as amblyopia, strabismus, or other ocular diseases from the records that could result in a misleading strabismus evaluation. Patients were excluded from the study if they underwent emergent operation for orbital wall fracture or had comorbidities with orbital wall fracture, such as traumatic optic neuropathy (cranial nerve II), loss of vision due to open globe injury or palsy of cranial nerve III, IV or VI.

According to the clinical practice protocol for orbital floor fracture, immediate management was suggested for patients if they presented with soft tissue entrapment with oculocardiac symptoms. This group of patients was excluded from analysis in this study. A regular operation for fracture repair was suggested for patients only if their condition fulfilled one of the following criteria: (a) diplopia persisting for longer than 7 days; (b) enophthalmos larger than 2 mm compared with the other eye; and (c) fracture size larger than 50% of the orbital floor. Based on the hospital protocol, ophthalmic consultation was recommended for all patients with periocular blunt injury with/without orbital wall fracture. A total of 141 cases were identified and included in our study.

**Figure 1:**
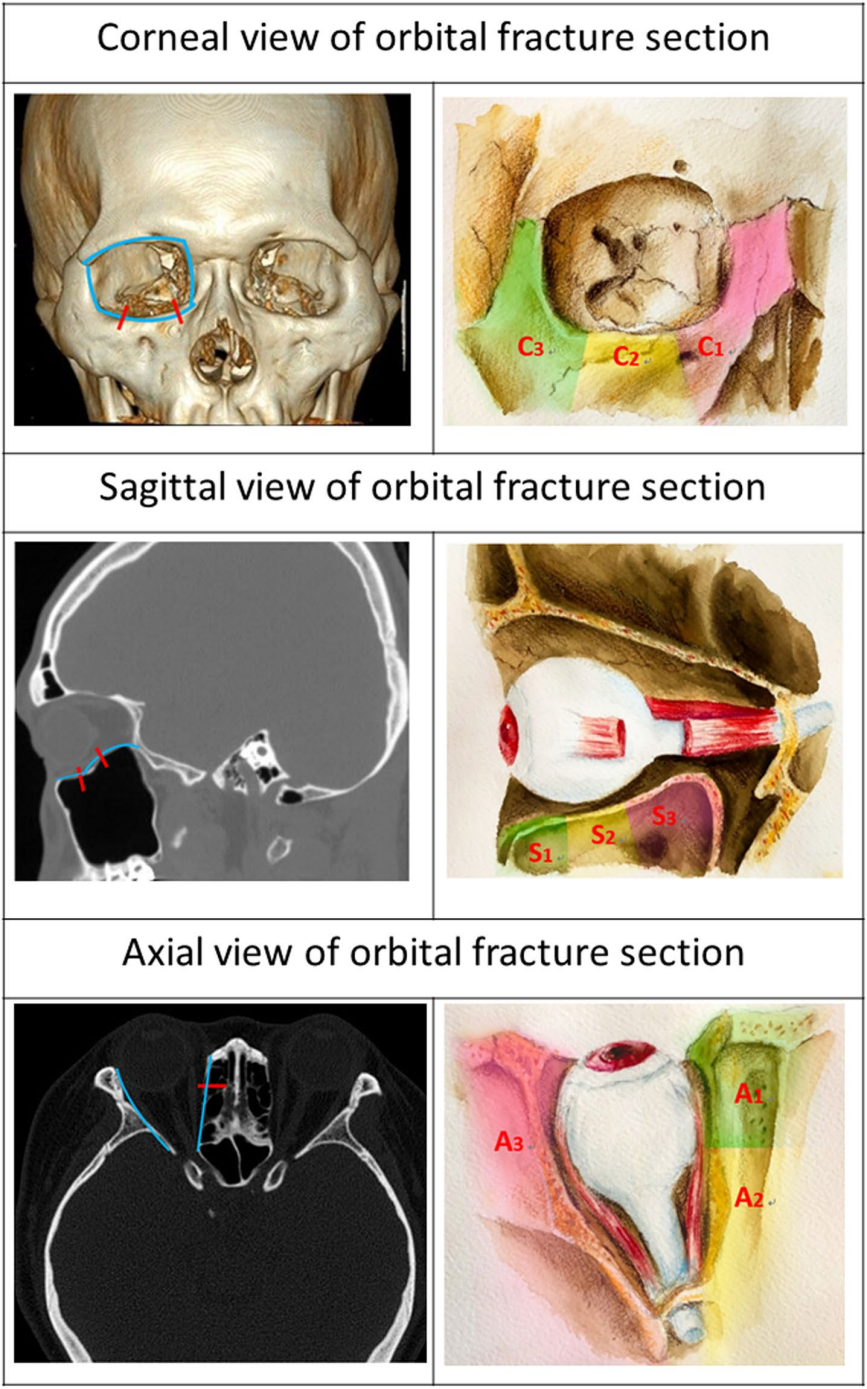
(Top left) Coronal view of a 3D-CT reconstruction. (Top right) Illustration of orbital floor fracture identified in the coronal view. (Middle left) Sagittal CT scan and (Middle right) illustration of orbital floor fracture identified in the sagittal view. (Bottom left) Axial CT scan and (Bottom right) illustration of orbital floor fracture identified in the axial view.

Detailed information from the medical records of each patient who underwent ophthalmologic examination during each follow-up visit was recorded. Data regarding best-corrected VA, extraocular muscle movement, detailed information regarding fractures and their management, and the follow-up period were collected for analysis. For strabismus evaluation, preoperative records were collected from an ophthalmic consultation before surgery, whereas postoperative strabismus was recorded at 3-month follow up after the operation. CT scans were routinely employed in all cases. One group of examiners reviewed sagittal, coronal and axial CT scans. In addition, orbital fractures identified in the coronal view were classified as either C1, C2 or C3, based on their location in the medial, middle and lateral walls (Fig. 1a,b). In the sagittal view, orbital fractures were classified as S1, S2 or S3, based on their location in the anterior, middle and posterior floor (Fig. 1c,d). In the axial view, orbital fractures were classified as A1, A2 or A3, based on their location in the anterior medial, posterior middle and lateral walls (Fig. 1e,f).

Data were analyzed using SPSS software version 18.0 for Windows (SPSS Inc., Chicago, IL, USA). All of the data for each group are presented as the mean (SD). We conducted Student’s t-test to compare the characteristics between the groups, and multiple regression analysis to examine predictive factors for postoperative strabismus. P < 0.05 was considered statistically significant.

## Results

From the eligible chart review, a total 141 adults (99 males and 42 females) met the inclusion criteria. The mean age (SD) of all enrolled patients was 49.7 (2.6) years old. The mean postoperative follow-up period (SD) was 6.2 (4.1) months. All patients were classified according to the presence or absence of diplopia in a primary position at a 3-month follow-up visit. In total, 28 patients (21 males and 7 females) belonged to the postoperative strabismus group (Group 1), and 113 patients (78 males and 35 females) belonged to the group without postoperative strabismus (Group 2). Regarding the laterality of injured eyes, the right eye was affected in 8 patients (20 in the left eye) in group 1, while the right eye was affected in 17 patients (96 in the left eye) in group 2. Among the causes of orbital wall fracture, the most common three causes were traffic accident (7, 25%), working (6, 21.4%) and sports (5, 17.9%) in group 1, whereas fighting (28, 24.8%), working (26, 23.0%) and traffic accident (24, 21.2%) were the top three causes in group 2. In addition to orbital wall fracture, associated injuries were common in these patients; traumatic optic neuropathy (without blindness) was the most common in group 1 (2, 7.1%), and facial trauma was the most common in group 2 (21, 18.9%). (Table 1) There were 98 patients with pre-operative diplopia and 28 patients with post-operative diplopia (PD).

**Table 1:** Baseline Characteristics of Patients who Underwent Repair of Orbital Wall Fracture.

	With PD (N = 28)	Without PD (N = 113)
**Gender**		
Male	21	78
Female	7	35
**Age**	51.2 (SD = 9.7)	47.5 (SD = 12.3)
**Laterality**		
Right eye	8	17
Left eye	20	96
**Causes of Fracture**		
Traffic accident	7	24
Fighting	2	28
Sports (or playing)	5	12
Working	6	26
Falling	2	9
NA	6	14
**Accompanying injury**		
TON	2	11
Open globe injury	1	2
Facial trauma	0	21
Traumatic cataract	0	2
Major trauma	1	4

PD = post-operative diplopia, NA = non-available, TON = traumatic optic neuropathy.

To determine which anatomical view of orbital wall fracture most commonly leads to post-operative diplopia (PD), we performed statistical analysis on the relationship between three sections of anatomical views and PD. According to the results, only the sagittal view section was significantly related to PD (p = 0.044) (Table 2). This result implied that patients with any fracture in the sagittal view of orbital CT may have a higher chance of sequalae involving PD than those with fractures in other views. Next, we aimed to determine whether fractures involving more than two section views of orbital CT resulted in a higher probability of PD. By analyzing all combinations of two or three sections, we found that only fractures involving both coronal and sagittal sections led to a significantly increased risk of PD (p = 0.031).

**Table 2:** Anatomical Factors related with residual diplopia after orbital walls fracture repair.

	With PD (N = 28)	Without PD (N = 113)	OR	*P* value
**Coronal Views**				0.396
C1	11	19	3.255	0.015*
C2	25	96	1.588	0.370
C3	8	19	2.092	0.093
**Axial Views**				0.139
A1	4	7	3.917	0.004*
A2	11	37	1.354	0.469
A3	8	42	0.730	0.297
**Sagittal Views**				0.044*
S1	8	10	2.528	0.050
S2	14	31	2.704	0.107
S3	11	17	3.778	0.006*

PD = post-operative diplopia, OR = odds ratio, *p < 0.05.

To examine the relationship between every single location of orbital wall fracture and the occurrence of postoperative strabismus according to a simplified anatomic scoring system, we performed statistical analysis and found that C1 (p = 0.015), A1 (p = 0.004) and S3 (p = 0.006) were significantly related to PD (Table 2).

Next, we wanted to determine if more than one location in every section of orbital wall fracture would result in a higher probability of PD. In the coronal section, C1 + C2 (p = 0.010), and C1 + C2 + C3 (p = 0.005), but not other combinations were significantly associated with PD. In the axial section, the A1 + A2 group (p = 0.034) and the A3 + A1 group (p = 0.005) were significantly related to PD. In contrast, S1 + S2 (p < 0.001), S2 + S3 (p = 0.006), and S1 + S2 + S3 (p < 0.001) in the sagittal section all significantly contributed to PD (Table 3).

**Table 3:** Anatomical Factors involving more than one locations leading to post-operative diplopia after orbital walls fracture repair.

	95% Confidence Interval		*P* value
	Lower	Upper	
**Fractures in Coronal Views**			
C1 + C2	−0.492	0.012	0.010*
C2 + C3	−0.297	0.157	0.261
C3 + C1	NA	NA	NA
C1 + C2 + C3	−0.187	0.027	0.005*
**Fractures in Axial Views**			
A1 + A2	-0.193	0.053	0.034*
A2 + A3	-0.202	0.142	0.503
A3 + A1	−0.187	0.027	0.005*
A1 + A2 + A3	−0.053	0.073	0.526
**Fractures in Sagittal Views**			
S1 + S2	−0.308	−0.012	<0.001*
S2 + S3	−0.408	0.028	0.006*
S3 + S1	NA	NA	NA
S1 + S2 + S3	−0.177	−0.003	<0.001*

*p < 0.05.

Using an anatomical scoring system based upon patients’ profiles, we aimed to determine whether any combination of fractures occurred more frequently and was correlated with PD. According to the results, fractures of C1 + A2 (Pearson correlation = 0.340, p < 0.001), C2 + S1 (Pearson correlation = 0.206, p = 0.031), A2 + A3 (Pearson correlation = 0.218, p = 0.022), A3 + S1 (Pearson correlation = 0.189, p = 0.048), A3 + S3 (Pearson correlation = 0.195, p = 0.041), and S2 + S3 (Pearson correlation = 0.449, p < 0.001) occurred more frequently than any other combinations and were correlated with PD. We suggest that this result reveals anatomical orbital structure correlates in the C1 + A2, C2 + S1, A3 + S1, A3 + S3 and S2 + S3 groups. However, fracture in the A2 + A3 group may be the result of increased traumatic force.

To make the anatomical scoring system more applicable to clinical practice, we aimed to determine the risk of PD in the presence of any orbital wall fracture. From odds ratio analysis in every location in this system, patients with fractures in C1 (odds ratio = 3.255), A1 (odds ratio = 3.917) and any S section (odds ratio = 2.528 in S1, 2.704 in S2 and 3.778 in S3) had more than a 2.5-fold chance of developing PD (Table 2).

Among all patients in our database, we also found that one special group of fractures, involving C2 + A3 and C3 + A3, zygomaticomaxillary complex fractures or tripod fractures, had no chance of developing PD. Atotal of 17 patients in our database belonged to this group, and based on ophthalmic records, none of these patients reported preoperative or postoperative diplopia. We attributed this finding to the attachment of all extraocular muscles related to strabismus to anatomical structures other than C3 and A3. In addition, this result may be used as a reference for surgical time determination in patients with tripod fractures.

## Discussion

Not every orbital floor fracture requires repair. The indications for orbital wall repair are as follows:

1. Entrapped muscle or periorbital tissue associated with a nonresolving oculocardiac reflex.
2. Symptomatic diplopia with positive forced ductions that persists unresolved for more than 7 to 10 days.
3. Large floor fracture (>50% floor area) causing latent or significant enophthalmos (>2 mm posterior displacement).

A few studies have demonstrated that diplopia after trauma will improve without surgery in some patients^2^. However, cases that resolved without surgery involved small orbital fractures and negative forced duction tests. Surgical repair is still required for the other 69.2% of patients with diplopia. Therefore, surgical treatment should not be delayed If patients meet the indications. Early Intervention decreases the risk that the impinged orbital tissues will develop fibrosis resulting in chronic diplopia^3^. Two recent case series reported improved surgical outcomes when surgery was offered within two weeks of injury^4,5^.

Diplopia is a common complication of orbital fractures. According to current studies, the incidence of diplopia caused by orbital fracture ranges from approximately 66.7% to 83%^3,6,7^. Ocular motility was reduced because of muscle or soft tissue entrapment^8^. Associated muscle edema, hemorrhage and motor nerve palsy can also lead to motility deficits. The goals of orbital surgery are to release the displacement and entrapment of ocular muscle or fat and recover extraocular motility.

However, the incidence of diplopia after the proper surgical repair of blowout fractures remains from 10 to 37%^9–13^. Persistent postoperative diplopia is related to many factors. Silbert and Matta^14^ reported that the most common causes of diplopia following the surgical repair of orbital floor fracture are as follows:

1. Incomplete removal of extraocular muscles and associated orbital tissue from the fracture site prior to implant placement.
2. Inappropriate placement of an implant, leading to orbital tissue entrapment.
3. Neuromuscular trauma.
4. Orbital adherence syndrome.

Careful assessment of patient symptoms and the location of orbital fractures (and the influence of related soft tissues) on CT is highly associated with surgical success. Tahiri and Lee^15^ reported that preoperative diplopia is the most important prognostic factor for the presence of postoperative diplopia after surgical repair. Avisham^3^ also reported that patients who exhibited preoperative diplopia were 3 times more likely to have persistent diplopia postoperatively. Therefore, preoperative diplopia may suggest that we should pay more attention to CT images to determine the location of the fracture and related soft tissue and neuromuscular injuries.

CT is the most common method used for the evaluation of orbital fractures. The location and size of the lesion and its effect on adjacent structures is clearly shown on CT.

Axial view: shows the fracture of the medial and lateral wall of the orbit.

Coronal view: demonstrates the fracture of the orbital floor.

Sagittal view: reveals the fracture of the orbital floor and posterior orbital apex.

Paolo^16^ reported that the most frequently involved orbital fracture site was the floor (80.3%), followed by the medial wall (9.2%) and lateral wall (1.1%). The incidence of orbital medial and lateral wall fractures was low. In addition, fracture of the lateral wall seldom causes diplopia. Significant associations were found between diplopia and eye elevation and orbital floor fractures (p < 0.05) and between horizontal diplopia and medial wall fractures (p < 0.05). These findings are consistent with those from our study, in which no postoperative diplopia (N = 17) was found for tripod fracture (C3 + A3) only.

Moreover, we found that the C1, A1 and S3 regions are significantly associated with postoperative strabismus in a single area fracture. Currently, patients with thyroid eye disease may receive orbital decompression, in which the orbital wall is broken, representing another form of orbital fracture. Previous studies^17,18^ showed that orbital decompression is much less likely to produce postoperative diplopia if performed on the “lateral wall” relative to the “floor” and “medial wall”. In addition, the inferomedial orbital strut, an important structure located in C1, separates the medial and inferior walls and provides structure to the bony orbital cone. Authors have argued that leaving the strut (or at least part of it) intact is vital to avoid hypoglobus and strabismus postoperatively^19–21^.

Irene and Mark presented several cases of posterior orbital floor fracture with inferior rectus muscle flap tear^22^. They hypothesized that the sudden downward force experienced by the orbital contents at the time of blunt trauma may exert traction on the connective tissue insertion into the orbital layer of the muscle, tearing the outer layer away from the inner, global layer. The implant is also easily placed too posteriorly when fractures are located in S3. More posteriorly placed implants pose a greater risk of crowding the orbital tissues and mechanically impeding movement of the extraocular muscles^23^.

These findings are consistent with those from our study, in which fractures over the C1, A1 and S3 regions were significantly associated with postoperative diplopia in a single area fracture.

Hawes and Dortzbach^24^ found that enophthalmos is more likely in cases of fractures involving more than half of the floor. In these cases, the inferior rectus, inferior oblique muscles, and inferior fibrofatty tissues can be easily contused, lacerated, or sheared by fractured bony fragments. Additionally, ocular motility can be restricted by subsequent muscular fibrosis and contraction^25^. These results correspond to our findings that fractures of more than one region in a single view (C, A, S) are significantly associated with postoperative diplopia. Thus, orbital fractures with greater soft tissue distortion or fractures involving more than half of the floor (even greater soft tissue distortion) should be operated on within 7 days.

Some results from the above-mentioned studies show that “floor” and “middle to posterior” orbital fractures are related to postoperative diplopia. This finding matches one of other our significant results; orbital fractures in the sagittal view are more often associated with postoperative diplopia in a single view. The coronal view can help us visualize an orbital floor fracture, whereas the sagittal view can help us to further evaluate a middle to posterior orbital floor fracture.

In our study, postoperative diplopia was also significantly associated with simultaneous fractures in sagittal and coronal views. This finding is consistent with Biesman’s^11^ study, in which fractures of the medial wall and floor combined seem to have a higher risk of leading to postoperative diplopia than fractures of the floor alone. Biesman suspected that the greater force required in cases of combined fracture might be distributed to orbital soft tissues.

In conclusion, our data confirm that orbital fractures found in the sagittal view are more often associated with postoperative diplopia in a single view. We also observed the C1, A1 and S3 regions are significantly associated with postoperative diplopia in a single area fracture. Fractures of more than one region in a single view (C, A, S) and simultaneous fractures in the sagittal and coronal views are significantly associated with postoperative diplopia. The results of this analysis can serve to remind physicians to pay more attention to the high-incidence area of diplopia before surgery for improved surgical success.
